# Survey dataset on the epidemiological assessment of cassava mosaic disease in South West and North Central regions of Nigeria reveals predominance of single viral infection

**DOI:** 10.1016/j.dib.2021.107282

**Published:** 2021-08-13

**Authors:** Angela O. Eni, Oghenevwairhe P. Efekemo, Olabode A. Onile-ere, Justin S. Pita

**Affiliations:** a Department of Biological Sciences, Covenant University, KM 10 Idiroko Road Ota, Nigeria; b Central and West African Virus Epidemiology Program, Covenant University Hub, Ota, Nigeria; c Laboratory of Plant Physiology, Université Felix Houphouët-Boigny (UFHB), 01 BPV 34 Abidjan 01, Côte d'Ivoire

**Keywords:** Cassava mosaic disease, Nigeria, Cassava, Food security, ACMV, EACMV, Epidemiology

## Abstract

The dataset presented here was collected during field surveys conducted in 2015 and 2017, to determine the distribution of African cassava mosaic virus (ACMV) and East African cassava mosaic virus (EACMV) across 12 Nigerian states and the Federal Capital Territory (FCT), Abuja. In each state, cassava farms were systematically sampled at 10 km intervals except in locations with sparse distribution of cassava farms. In each farm, 30 cassava plants were visually assessed for presence or absence of cassava mosaic disease (CMD) foliar symptoms along two diagonals. Whitefly population was assessed by counting the number of whiteflies on the top five leaves of each sampled plant. Then an average of 4 cassava leaf samples were collected from each farm, and screened for ACMV and EACMV infections using polymerase chain reaction. The dataset includes CMD incidence, symptom severity and the relative abundance of whiteflies in each field as well as laboratory results that show the distribution of ACMV and EACMV across the regions surveyed.

## Specifications Table

**Table:** 

Subject	Agricultural and Biological Sciences
Specific subject area	Survey of cassava mosaic begomoviruses
Type of data	Table Figure Code in Jupyter notebook
How data were acquired	Data was collected during field surveys conducted in 2015 and 2017 across the South West and North Central regions of Nigeria. 30 cassava plants were sampled in cassava farms located along interstate road networks. An average of 4 cassava leaf samples were collected from each farm and analysed for the presence of ACMV and EACMV using polymerase chain reaction
Data format	Raw Analysed Filtered
Parameters for data collection	Symptom assessment on the field Whitefly assessment on the field Nucleic acid amplification of virus genes
Description of data collection	Data was collected as part of surveys of cassava mosaic begomoviruses in study region
Data source location	City/Town/Region: *South West and North Central Nigeria* Country: *Nigeria*
Data accessibility	http://dx.doi.org/10.17632/mpj2nxk3tk.1
Related research article	Eni, A. O., Efekemo, O. P., Onile‐ere, O. A., & Pita, J. S. (2020). South West and North Central Nigeria: Assessment of cassava mosaic disease and field status of African cassava mosaic virus and East African cassava mosaic virus. *Annals of Applied Biology*, (September), aab.12647. https://doi.org/10.1111/aab.12647

## Value of the Data

• The two years field survey data presented here provides an update on the distribution of cassava begomoviruses in Nigeria since the last surveys conducted over ten years ago.
• The data presented here is useful to governments and agricultural stakeholders who need to plan and implement interventions towards the management of cassava begomoviruses in Nigeria.
• The data presented here could serve as baseline for future endeavours at mapping the distribution of cassava mosaic begomoviruses.
• Data could be used to model disease spread pattern.

## 1 Data Description

**Fig. 1:**
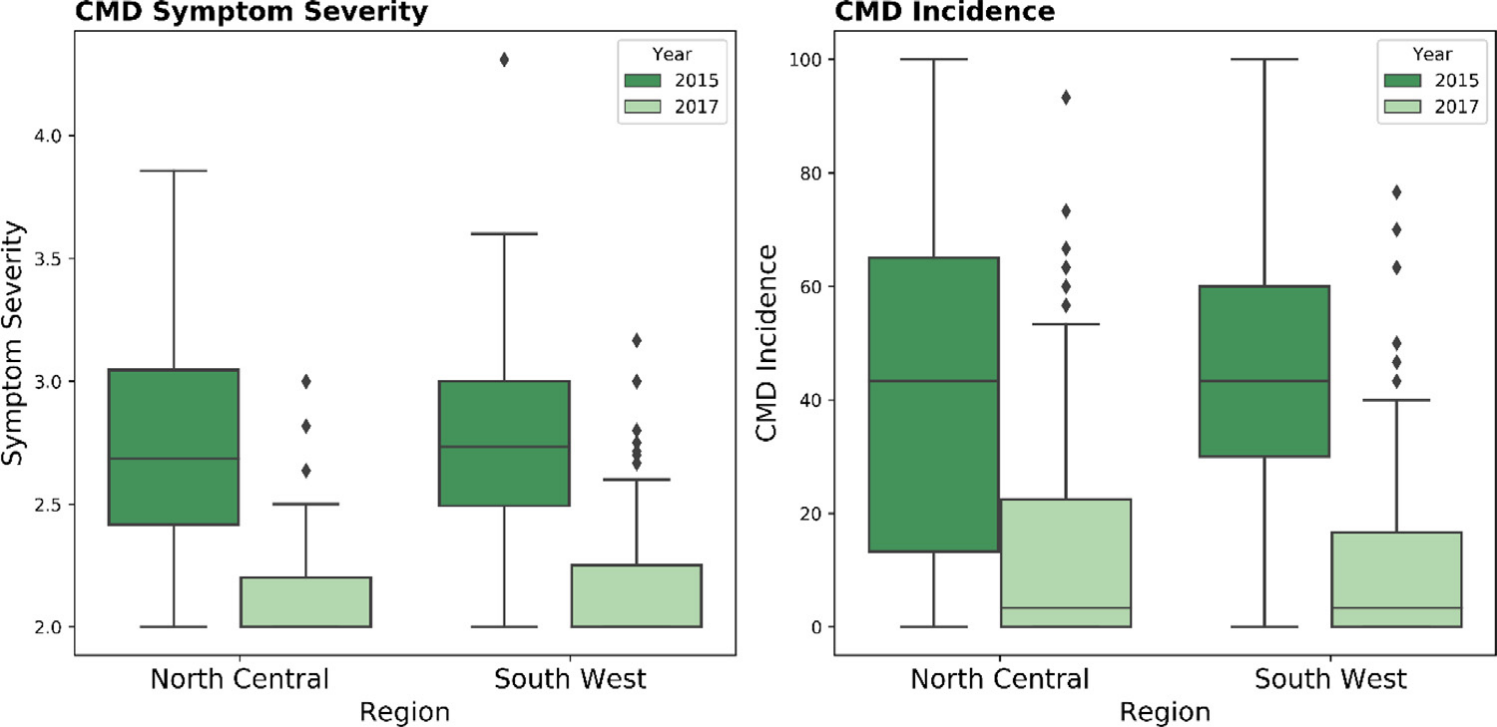
CMD incidence and CMD symptom severity in the North Central and South West Nigeria in 2015 and 2017.

The dataset provided with this submission contains field and laboratory results of samples collected during surveys conducted in 2015 and 2017 across the South West and North Central regions of Nigeria (Fig. 1). A total of 184 and 328 cassava farms were surveyed in 2015 and 2017, respectively from which 613 and 704 cassava leaf samples were collected and analysed (Table 1).

**Table 1:** Number of fields surveyed per state.

	2015		2017	
	Count	%	Count	%
Benue	30	16.3%	34	10.4%
Ekiti	11	6.0%	20	6.1%
FCT	1	0.5%	2	0.6%
Kogi	16	8.7%	32	9.8%
Kwara	12	6.5%	20	6.1%
Lagos	3	1.6%	9	2.7%
Nasarawa	10	5.4%	30	9.1%
Niger	13	7.1%	13	4.0%
Ogun	28	15.2%	36	11.0%
Ondo	15	8.2%	39	11.9%
Osun	12	6.5%	32	9.8%
Oyo	24	13.0%	50	15.2%
Plateau	9	4.9%	11	3.4%
Total	184	100.0%	328	100.0%

### 1.1 Dataset documentation

The dataset is provided as long form tables in an excel file with three worksheets as contained in Table 2. Variable information for each worksheet is provided in Table 3.

**Table 2:** Details of worksheets in the provided dataset.

Sheet Name	Info
Field	Contains data collected on the field such as location, CMD symptom severity and CMD incidence
Lab	Contains data for each sample analysed
Field_Lab	Contains laboratory data aggregated by field

**Table 3:** Description of variables contained in the dataset provided.

Variable Name	Description	Scale Type	Categories
**Field Worksheet**			
Year	Year of survey	Binary	2015, 2017
Field	Investigator assigned field number. Column could be used as id column to merge data from other worksheets	Numeric	-
Country	Country of survey. Constant- Nigeria	-	-
State	State	Nominal	Benue, Ekiti, FCT, Lagos, Kogi, Kwara, Ogun, Nasarawa, Ondo, Osun, Oyo, Niger, Plateau
Altitude	Altitude in meters	Numeric	-
Mean_CMD_Severity	CMD symptom severity as observed and scored on the field. Scoring rubric is in the methods section. This variable was calculated as follows. $\text{Mean}\;\text{CMD}\;\text{Severity}=\frac{\sum scores\;for\;plants\;showing\;symptoms}{total\;number\;of\;plants\;showing\;symptoms}$	Numeric	-
CMD_Incidence	$Incidence\;\left(\%\right)=\frac{Number\;of\;plants\;showing\;symptoms}{Total\;number\;of\;sampled\;plants\;in\;a\;field}*100$	Numeric	-
Cutting_Infection	Percentage of infections deemed as originating from the propagation of infected cassava stem cutting. See methods section. In a field, the summation of the proportion of whitefly infections and cutting infections would always equal 1 (or 100%). Blank where there are no plants showing signs of infection	Numeric	-
Whitefly_Infection	Percentage of infections deemed as originating from the whitefly vector transmission. See methods section. In a field, the summation of the proportion of whitefly infections and cutting infections would always equal 1 (or 100%). Blank where there are no plants showing signs of infection	Numeric	-
Total_whitefly	Total number of whiteflies counted as described in methods section	Numeric	-

**Lab Worksheet**			
Year	Year of survey	Binary	2015, 2017
Host	Point of collection; whether sample was collected from a cassava plant or from another plant species (mostly weeds) showing the characteristic mosaic symptoms	Binary	Cassava, Alternate host
Zone	Geopolitical zone of sampling location South West States include – Ekiti, Lagos, Ogun, Ondo, Osun and Oyo North Central States include – Benue, FCT, Kogi, Kwara, Nasarawa, Niger, Plateau * FCT is technically not a state, it is the capital of Nigeria, however in this dataset it is treated as such	Binary	South West, North Central
State	State	Nominal	Benue, Ekiti, FCT, Lagos, Kogi, Kwara, Ogun, Nasarawa, Ondo, Osun, Oyo, Niger, Plateau
Field	Investigator assigned field number. Column could be used as id column to merge data from other worksheets	Numeric	-
Sample No	Investigator assigned ID variable	Numeric	-
Severity_Score	CMD symptom severity of sampled plant. Note- Severity score of 1 implies a plant not showing symptoms as explained in the methods section	Numeric	-
ACMV	Binary variable for whether virus is present in sample	Binary	0-Absent 1-Present
EACMV	Binary variable for whether virus is present in sample	Binary	0-Absent 1-Present
Mixed	Binary variable for whether sample contains a mixed infection	Binary	0-Absent 1-Present
EACMCV	East African cassava mosaic Cameroon virus- Results only available for samples positive for EACMV	Binary	1- Present
Symptom	Binary variable for whether plant showed symptom	Binary	0-Asymptomatic 1- Symptomatic
Result	Result for sample	Nominal	ACMV, EACMV, Mixed, Negative

Field_Lab Worksheet* laboratory results for non-cassava hosts not included in this aggregate			
Year	Year of survey	Binary	2015, 2017
State	State	Nominal	Benue, Ekiti, FCT, Lagos, Kogi, Kwara, Ogun, Nasarawa, Ondo, Osun, Oyo, Niger, Plateau
Field	Investigator assigned field number. Column could be used as id column to merge data from other worksheets	Numeric	-
ACMV	Number of samples with ACMV infection in field	Numeric	
EACMV	Number of samples with EACMV infection in field	Numeric	
Mixed	Number of samples with mixed infection in field	Numeric	
Negative	Number of unreactive (negative)	Numeric	

Result	Aggregated laboratory results by field. There are 5 possible outcomes here **ACMV** – A field in which only ACMV is found to be infecting plants **EACMV** - A field in which only EACMV is found to be infecting plants **EACMV+ACMV** – A field in which both EACMV and ACMV occur but not as a mixed infection. i.e both viruses singly infecting plants in one field **Mixed –** A field in which ACMV and EACMV are found to be infecting the sample plant. **Negative –** Fields without any infected plants *because it is possible to have multiple possibilities in a field. The results are decided based on the following hierarchy Mixed> EACMV+ACMV> EACMV> ACMV> Negative		ACMV, EACMV, EACMV+ACMV, Mixed, Negative

### 1.2 Exploration of dataset

Here we present an exploration of the dataset, all codes used for this exploration are available as a supplementary python script and Jupyter notebook.

a. *CMD incidence and CMD symptom severity*

Summary of CMD incidence and CMD symptom severity in the different regions across 2015 and 2017 is presented in Fig. 1.

b. *Origin of infection and whitefly abundance*

**Fig. 2:**
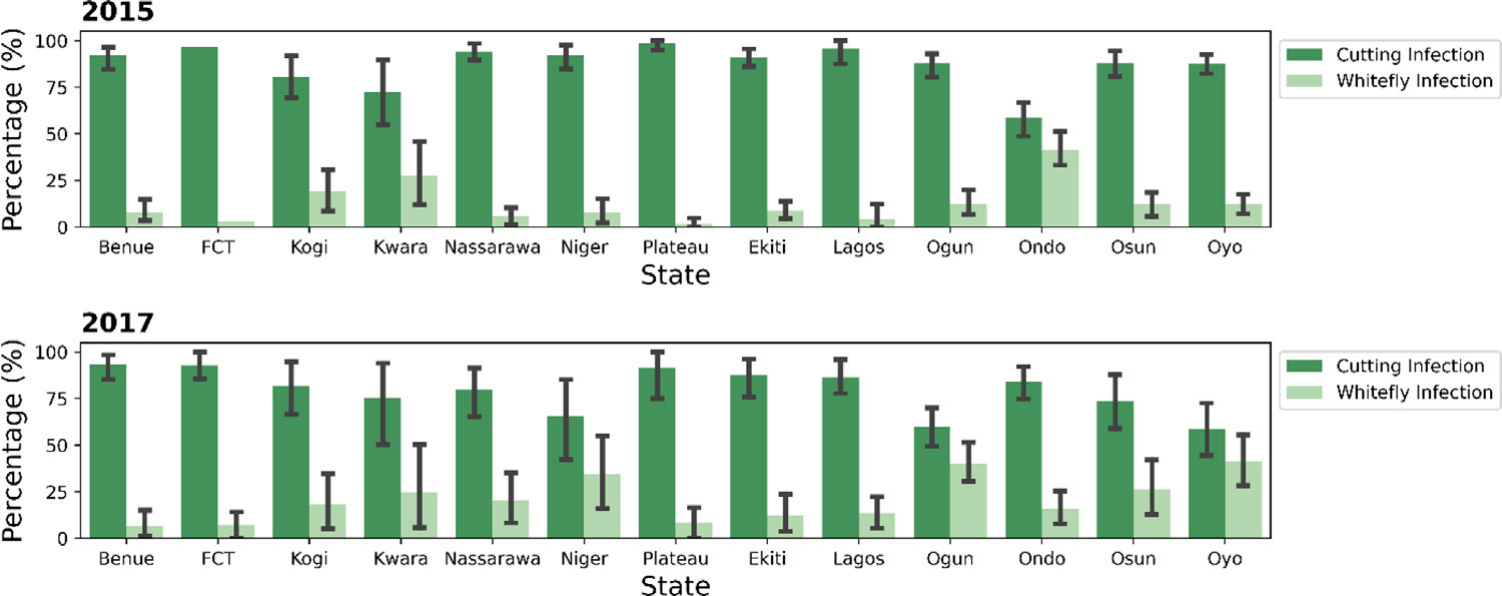
Proportion of cutting transmitted and whitefly transmitted CMD infections across States in North Central and South West Nigeria surveyed in 2015 and 2017.

**Fig. 3:**
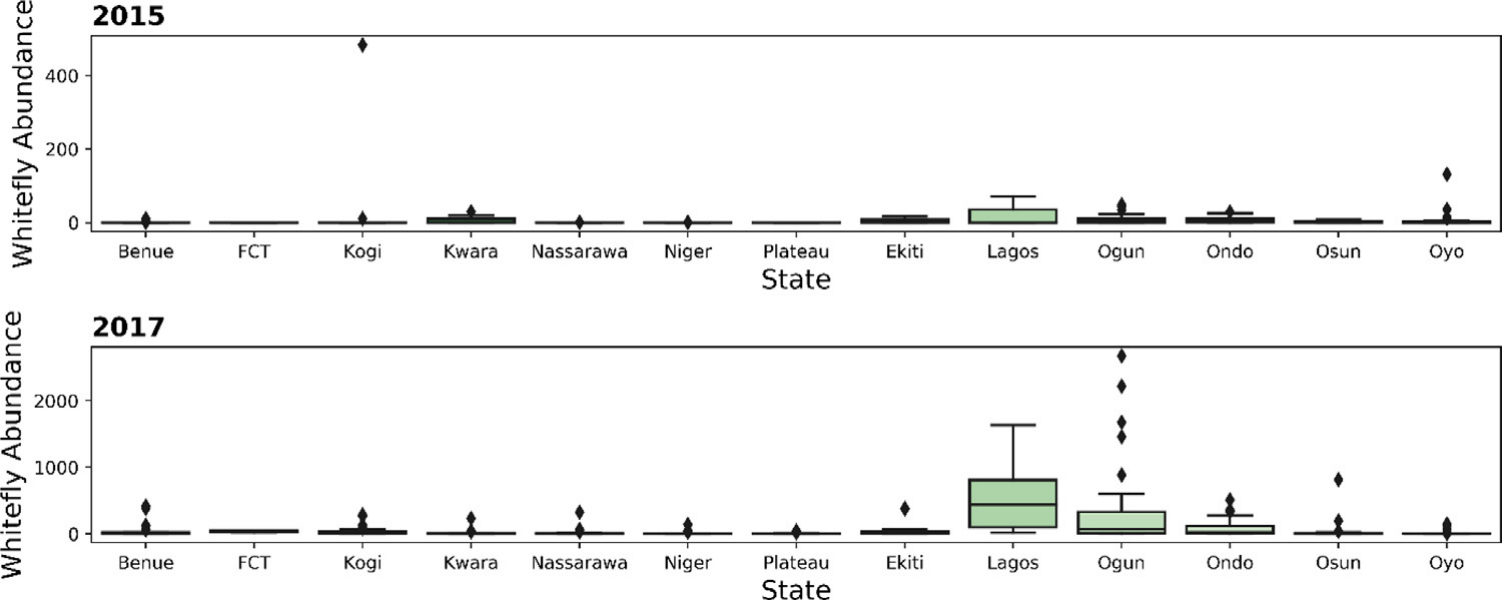
Relative whitefly abundance across States in North Central and South West Nigeria surveyed in 2015 and 2017.

Summary plot showing the proportion of infections originating from whitefly vector transmission versus infections as a result of the propagation of infected cuttings is presented in Fig. 2. Summary plots for whitefly abundance across the states surveyed is presented in Fig. 3.

c. *Type of Begomovirus infection*

**Fig. 4:**
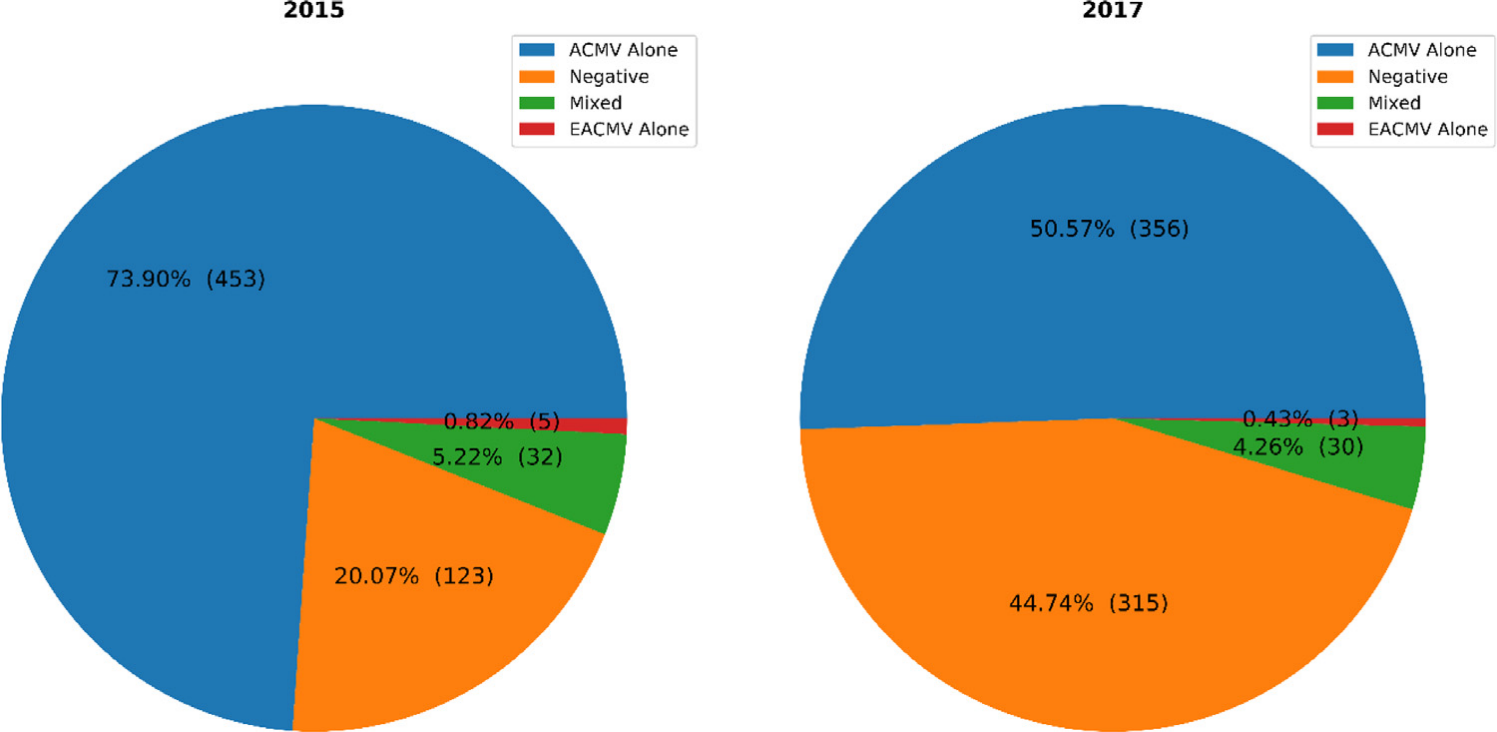
Proportion of ACMV infected, EACMV infected, mixed ACMV & EACMV infected and uninfected cassava leaf samples across North Central and South West Nigeria in 2015 and 2017.

**Fig. 5:**
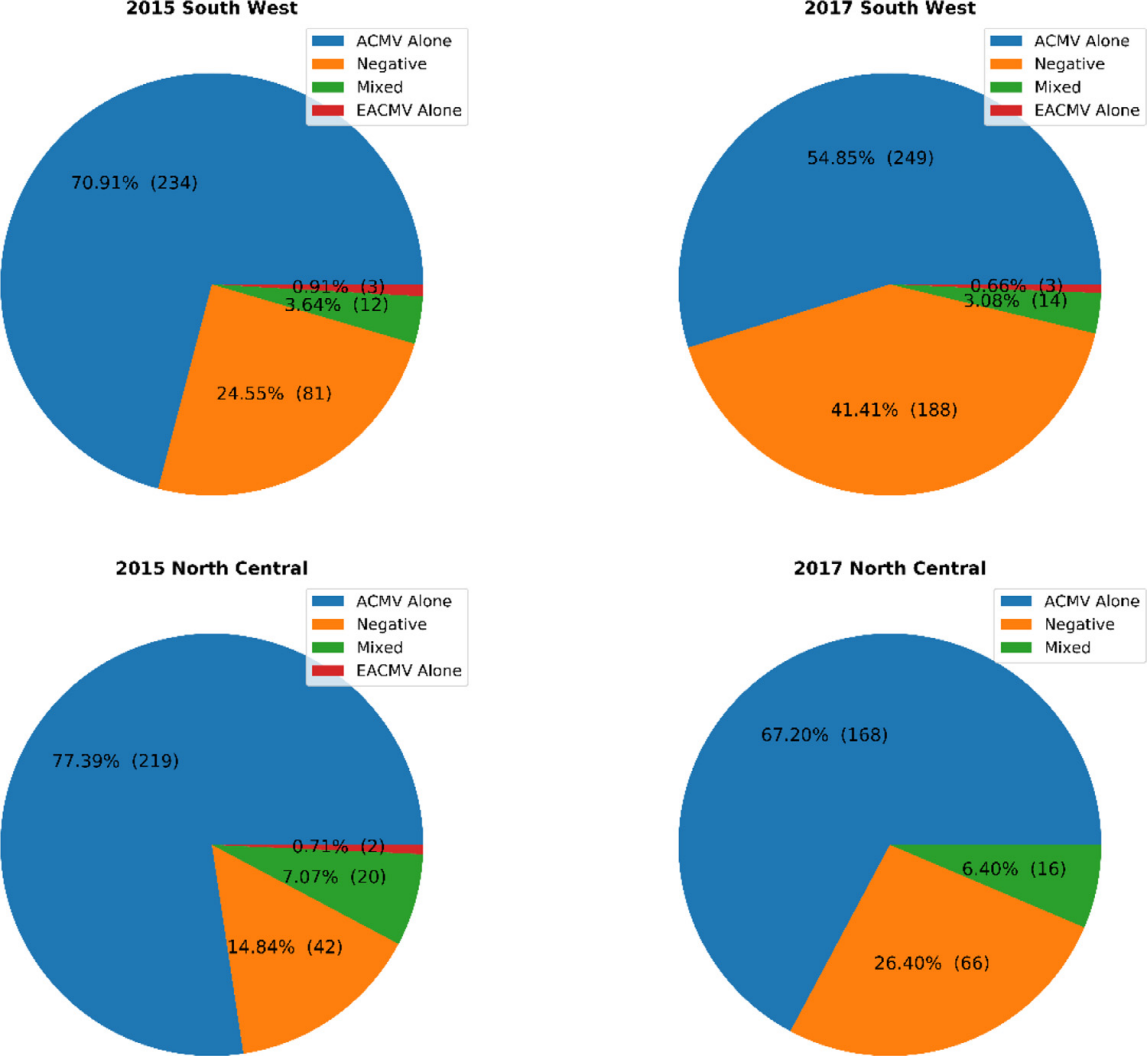
Proportion of ACMV infected, EACMV infected, mixed ACMV & EACMV infected and uninfected cassava leaf samples in the North Central and South West regions in 2015 and 2017.

All samples collected were assessed for the presence of ACMV or EACMV by PCR. Samples were either negative, positive for either virus or positive for both viruses in a mixed infection. Summary plots on the proportion of the different viruses in collected samples are presented in Fig. 4, Fig. 5.

## 2 Experimental Design, Materials and Methods

### 2.1 Survey

We conducted surveys of cassava farms across the South West and North Central regions of Nigeria in 2015 and 2017.

### 2.2 Sampling

Sampling was performed following previously described methods with slight modifications [1]. Following a road map of the surveyed regions, cassava farms located at an average of 10 km apart along surveyed routes were sampled. In each farm, 30 cassava plants were randomly selected along two diagonals and observed for the presence or absence of CMD symptoms. For plants exhibiting CMD symptoms, symptom severity was scored following previously described methods [2]. CMD symptom severity was scored on a scale of 1–5 as previously described [3,4]. CMD incidence was calculated as the proportion of sampled plants showing CMD symptoms. For symptomatic plants, the origin of the infection was determined based on the distribution of symptoms on the plant as previously described [2,5]. The relative abundance of whitefly vectors in each farm was determined by counting the number of whiteflies present on the underside of the five topmost leaves of each of the 30 plants sampled within the farm. Then an average of four (4) cassava leaf samples were collected and stored in herbarium presses prior to laboratory analysis.

## 3 Molecular Detection of Cassava Mosaic Begomoviruses

### 3.1 DNA extraction

Extraction of DNA was carried out following the methods of Dellaporta et al. [6]. The concentrations of the extracted DNA were assessed using Nano Drop 2000 spectrophotometer (Thermo Fisher Scientific, Waltham, Massachusetts, USA) and adjusted to 50 ng/µl for PCR.

### 3.2 PCR

The isolated DNA were screened for ACMV and EACMV by polymerase chain reaction according to the methods of Fondong et al. [7]. Multiple specific PCR primers were used to ensure that strain variations were adequately captured (Table 4). The PCR mixture contained 1 × PCR reaction buffer [200 mM Tris HCl (pH 8.4) and 500 mM KCl], 10 mM dNTPs (Promega, Madisson Wisconsin USA), 25 mM MgCl2, 20 pmol of each primer and 1 U of Taq DNA Polymerase (Promega). The PCR products were resolved on a 1% agarose gel stained with ethidium bromide (10 mg/ml) alongside a 1 kbp plus DNA ladder (Thermo Fisher Scientific) at 100 V. The gels were analysed under UV light using a gel documentation system (UVP Gel Doc-IT2, LLC Analytik Jena, Germany).

**Table 4:** List of Primers used in detecting Cassava mosaic begomoviruses.

Primer Pair	Specificity	Primer Sequence	Reference
JSP 1 & 2	ACMV	ATGTCGAAGCGACCAGGAGAT	[8]
		TGTTTATTAATTGCCAATACT	
JSP 1 & 3	EACMV	ATGTCGAAGCGACCAGGAGAT	[8]
		CCTTTATTAATTTGTCACTGC	
ACMVB F&R	ACMV	TCGGGAGTGATACATGCGAAGGC	[9]
		TCGGGAGTGATACATGCGAAGGC	
EACMV 1 & 2	EACMV	GTTCGGCTATCACCTTCTAGAACA	[9]
		CAAGGCTTACATTGAAAAGGGA	
EAB555 F & R	EACMV	TACATCGGCCTTTGAGTCGCATGG	[10]
		CTTATTAACGCCTATATAAACACC	
VNF031/F & VNF032/R	EACMCV	GGATACAGATAGGGTTCCCAC	[10]
		GACGAGGACAAGAATTCCAAT	

## CRediT Author Statement

**Angela O. Eni** Conceptualisation, Methodology, Funding acquisition, Writing – review & editing; **Oghenevwairhe P. Efekemo:** Investigation; **Olabode A. Onile-ere:** Original draft preparation, Formal analysis; **Justin S. Pita:** Conceptualisation, Methodology, Funding acquisition.

## Declaration of Competing Interest

The authors declare that they have no known competing financial interests or personal relationships which have, or could be perceived to have, influenced the work reported in this article.
